# Genomic, virologic, and epidemiologic surveillance to track intrafamilial Mpox, Brazil

**DOI:** 10.1128/spectrum.03701-25

**Published:** 2026-04-22

**Authors:** Adriana de Souza Andrade, Mariella Sousa Coêlho Maciel, Ana Gabriella Stoffella-Dutra, Silvia Hees de Carvalho, Aline Almeida Bentes, Paulo Emilio Tonaco Costa, Iago José da Silva Domingos, Pedro Henrique Bastos e Silva, Talita Émile Ribeiro Adelino, Felipe Campos de Melo Iani, Luiz Carlos Júnior Alcantara, Giliane de Souza Trindade, Erna Geessien Kroon, Mauricio Teixeira Lima, Marco Antônio Campos

**Affiliations:** 1 Fundação Oswaldo Cruz, Instituto René Rachou 154611 , Belo Horizonte, Minas Gerais, Brazil; 2 Instituto Nacional de Ciência e Tecnologia em Poxvírus, Belo Horizonte, Brazil; 3 Universidade Federal de Minas Gerais - Departamento de Microbiologia 28114, Belo Horizonte, Minas Gerais, Brazil; 4 Hospital Eduardo de Menezes, Fundação Hospitalar do Estado de Minas Gerais - Serviço de Doenças Infecciosas e Parasitárias 186064, Belo Horizonte, Minas Gerais, Brazil; 5 Universidade Federal de Minas Gerais - Departamento de Pediatria 28114, Belo Horizonte, Minas Gerais, Brazil; 6 Fundação Hospitalar do Estado de Minas Gerais - Hospital Infantil João Paulo II, Belo Horizonte, Minas Gerais, Brazil; 7 Fundação Ezequiel Dias - Instituto Octávio Magalhães, Belo Horizonte, Minas Gerais, Brazil; Victorian Infectious Diseases Reference Laboratory, Melbourne, Australia

**Keywords:** Mpox, *Orthopoxvirus*, disease transmission, household, isolation, whole genome sequencing

## Abstract

Here, we report a fatal case of *Orthopoxvirus monkeypox* (MPV) infection in an immunocompromised patient with advanced HIV/AIDS, syphilis, and methicillin-resistant *Staphylococcus aureus* co-infection during the 2022 mpox epidemic in Brazil. Clinical, serologic, molecular, viral isolation, and genomic investigations revealed intrafamilial transmission within a multigenerational household, including older women and children. Notably, a 3-year-old child without direct contact with the index case developed clinical signs of mpox and tested positive for MPV by PCR and viral isolation. Genomic sequencing and phylogenetic analyses confirmed the B.1.9 lineage and showed that all viral sequences clustered within the same transmission chain. Serologic assays detected OPV-neutralizing antibodies in an older woman. These findings underscore the potential for prolonged MPV replication and household spread beyond traditionally recognized risk groups, emphasizing the need for strengthened genomic and epidemiologic surveillance, particularly for immunocompromised individuals and their families and caregivers.

**IMPORTANCE:**

Mpox expanded globally in 2022, but pediatric and household transmission remains poorly defined. We report a fatal infection in an immunocompromised patient with advanced HIV/AIDS during the epidemic in Brazil. Integrated clinical and genomic investigations revealed intrafamilial spread within a multigenerational household, including a 3-year-old child who had no direct contact with the index case. Viral genomes clustered in lineage B.1.9, and neutralizing antibodies were detected in an older woman and domestic animals. These findings highlight the potential for household transmission beyond recognized risk groups and underscore the need for strengthened surveillance.

## INTRODUCTION

Mpox is a zoonotic disease caused by *Orthopoxvirus monkeypox* (MPV), a member of the *Orthopoxvirus* (OPV) genus (1). Formerly, it was endemic in Central and West Africa (2), but it spread globally in 2022, with transmission largely among adult men. Since Clade IIb began circulating worldwide in 2022, the World Health Organization has reported 150,889 confirmed cases as of 30 June 2025 (3). Usually, self-limited mpox has a 6- to 16-day incubation and symptoms lasting 2–4 weeks. Common signs include abrupt fever, headache, myalgia, back pain, chills, asthenia, and lymphadenopathy. Skin lesions typically appear 1–3 days after symptom onset and evolve through macule, papule, vesicle, pustule, and crust stages; mucosal involvement can also occur (4, 5).

Although mpox typically follows a predictable clinical pattern, severity and course can vary, particularly during new outbreaks. In Brazil, we report an October 2022 case that resulted in death early in the epidemic when the first local cases were being identified (6). Given the still-evolving clinical and epidemiological profile, studies detailing symptoms, differential diagnosis, and transmission are essential to guide appropriate management.

Understanding mpox transmission is also critical for its containment. The disease is a zoonosis with an unidentified reservoir, notwithstanding tropical African rodents (e.g., tree squirrels, Gambian pouched rats) are prime candidates, and African non-human primates may act as intermediate hosts. Multiple mammals, including rabbits, prairie dogs, and other rodents, are susceptible in captivity and laboratory settings (5). Because MPV is pantropic, circulation in non-endemic regions raises concern for new endemic foci. Human-to-human spread occurs mainly through close contact with respiratory droplets, skin lesions, or contaminated objects, typically requiring physical proximity (4, 5, 7). In Brazil and worldwide, most cases occur in men around 30 years old and are frequently associated with other sexually transmitted infections (8, 9).

Mpox is transmitted mainly via close person-to-person contact with skin lesions, contaminated objects, or, less often, respiratory secretions (10). In the UK, the estimated household secondary attack rate is 4% (11). Pediatric cases are rare, and the transmission dynamic remains unclear. In November 2022, California reported 5,572 cases, which represented 20% of U.S. totals, yet only 0.3% were in individuals under 16 years old (12). Children living with infected adults may face higher risk. One study showed a 4.7% secondary attack rate in households with 129 exposed children. This rate is likely underestimated, as many of the children were either untested or exhibited mild symptoms (12).

Intrafamilial transmission has been documented in various settings. In the Central African Republic (2018), the transmission occurred within households beyond healthcare exposures (13). A family cluster imported from Nigeria to the UK in 2021 showed home transmission risk (14). More recently, a French report described infection of two parents and two children, whose mild presentations might have been missed without known exposure (15).

Here, we report intrafamilial transmission of mpox within an ecologically rich household comprising 11 adults and 3 children. To support a better understanding of the risk and clinical presentation in exposed pediatric cases, genomic data corroborate clinical evidence of linked MPV infections.

## MATERIALS AND METHODS

### Clinical data and biological sample collection

Collected materials included nasal scabs, nasal vesicle fluid, pustules (including abdominal), saliva, nasal swabs, and oropharyngeal swabs. Patients were admitted to Hospital Eduardo de Menezes (HEM) and Hospital Infantil João Paulo II, both in Belo Horizonte, Minas Gerais, Brazil. Selected samples came from members of the same household who had close contact.

### Cells and viruses

Vero and BSC-40 cells were used for viral replication and plaque reduction neutralization tests (PRNT), respectively. Cells were cultured in high-glucose Dulbecco’s modified Eagle medium (DMEM) (Millipore Sigma, MA, USA) supplemented with 5% fetal bovine serum (FBS) (Gibco, Thermo Fisher Scientific, NY, USA) and 1% Penicillin-Streptomycin (Gibco, Thermo Fisher Scientific, NY, USA). All cells were maintained at 37°C, 5% CO₂, and 95% humidity. The Western Reserve of orthopoxvirus vaccinia (VACV-WR) used in this study was kindly provided by the Laboratório de Vírus at the Universidade Federal de Minas Gerais (UFMG), Belo Horizonte, Minas Gerais, Brazil. The virus was replicated in Vero cells at the Laboratório de Imunologia das Doenças Virais, FIOCRUZ-MINAS, Belo Horizonte, Minas Gerais, Brazil, and titrated in BSC-40 cells, yielding a titer of 10⁶ plaque-forming units (PFU) per mL.

### Virus isolation

Isolation followed a published protocol with adaptations (16). All work occurred in a BSL-3 lab (UFMG, Belo Horizonte, Brazil) in accordance with MPV biosafety procedures. Clinical samples were processed in PBS with amphotericin B (20 μg/mL), penicillin (1,000 U/mL), and streptomycin (500 μg/mL); serially diluted (1:10, 1:50, 1:100, 1:1,000, 1:10,000); vigorously mixed; subjected to three −20°C freeze–thaw cycles; centrifuged (2,000 *g*, 3 min); supernatants collected. Vero cells (ATCC CCL-81) were seeded in 25-cm² flasks (1.5 × 10⁶ cells) or six-well plates (6 × 10⁵ cells) and infected with 600 μL of processed supernatant. Cultures were incubated at 37°C, 5% CO₂ until cytopathic effect; flasks were gently mixed every 10 min for 1 h; a negative control was included. Monolayers were examined daily by light microscopy. After 48–72 h, virus was passaged similarly, up to a third passage.

### Molecular screening

After MPV isolation, genome detection was performed by qPCR. For all samples undergoing isolation, culture supernatants were collected and tested. DNA was extracted from 200 μL of each supernatant using the High Pure Viral Nucleic Acid Kit (Roche, Penzberg, Germany) per manufacturer. TaqMan qPCRs targeting the viral DNA polymerase and TNF receptor genes were run as previously described (17). A clade II MPV strain from the 2022 outbreak, provided by the UFMG Virus Laboratory (Brazil), served as the positive control.

### PRNT

Neutralizing titers were measured by PRNT with VACV-WR (16). Sera were heat-inactivated (56°C, 30 min), serially diluted, and incubated with 200 PFU VACV for 18 h. Confluent BSC-40 monolayers (6-well) were infected with 300 µL serum–virus mix, incubated 1 h at 37°C, and then overlaid with 2 mL DMEM + 2% FBS. Plates incubated at 37°C, 5% CO₂, 95% humidity. After 48 h, monolayers were fixed with 3.7% formalin for 2 h and stained with crystal violet for 20 min. Plaques were counted, and the percentage of reduction was calculated vs virus control. PRNT₅₀ was the highest serum dilution reducing plaques ≥50%.

### Genome assembly and phylogenetic analysis

Viral DNA was amplified using MPV-specific amplicon-tiling primers, as previously described (18), and sequenced on the Illumina MiSeq platform according to the manufacturer’s instructions (Illumina, San Diego, CA, USA). Genomes were assembled with Minimap2 v2.24 and manually curated (19). To confirm the genotype, we downloaded Brazilian MPV Clade IIb genomes from 2022 to 2023 without GISAID warnings (accessed December 2024; *n* = 233). We re-evaluated them in Nextstrain v3.15.3 and excluded sequences with anomalous insertions or excessive mutations, yielding 209 genomes (Table S1) (20, 21). For B.1.9, 104 warning-free GISAID genomes (accessed July 2025) were similarly screened, aligned, and pruned by preliminary trees, leaving 80 genomes (Table S2) (20, 21). Both data sets were aligned with MAFFT v7.450, and Maximum Likelihood trees were inferred in IQ-TREE 2 (2.4.0) with 1,000 bootstraps (22, 23). ModelFinder selected the best-fit substitution model (24). Trees were visualized/edited in iTOL v7 (25). Genotyping used Nextclade v3.15.3 (Mpox virus [Clade IIb]), which also supported mutation comparisons (21).

## RESULTS

### Index case

**Fig 1:**
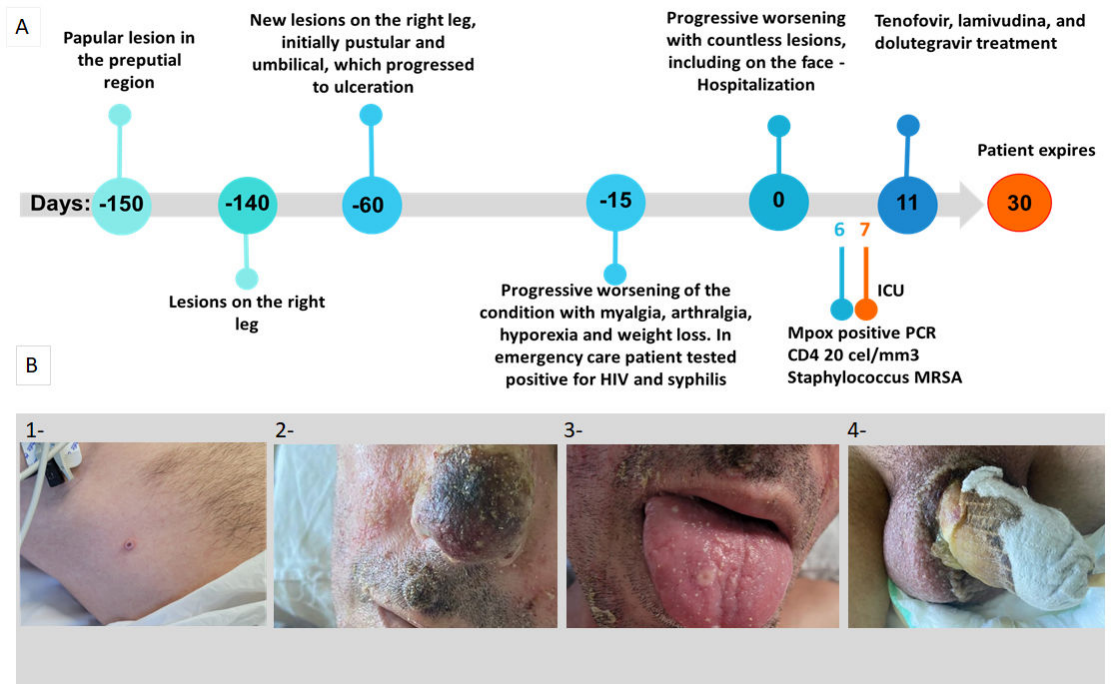
Timeline of clinical course and cutaneous lesions in the index case. (**A**) Timeline of symptoms, lesion sites, key clinical events, and sampling dates. (**B**) Photographs of representative lesions on day 7.

A 41-year-old man reported multiple unprotected partners of both sexes, and their last contact was 10 days before the first lesion (–160 days) (Fig. 1A). A papular preputial lesion appeared (–150 days). Around 10 days later (–140 days), a right lower-limb lesion impaired gait, followed by additional lesions and left upper-limb edema. Later (–60 days), new right-leg lesions developed with progressive myalgia, arthralgia, hyporexia, and weight loss. He sought emergency care shortly before admission (–15 days). Rapid HIV and syphilis tests were positive. He was admitted to HEM (day 0), prior to initiation of antiretroviral therapy (ART); on admission, he was pale, afebrile, and without lymphadenopathy, with crusted facial (Fig. 1, panel B1) and oral lesions (Fig. 1, panel B2), dysuria, and constipation.

On day 1, necrotic penile tissue was debrided with muscle preserved. On day 6, MPV PCR from skin scraping and an abdominal-lesion swab was positive (Fig. 1, panel B4).

Labs showed leukocytosis with lymphopenia (14,300/990 cells/mm³, respectively) and CD4 20 cells/mm³; culture grew methicillin-resistant *Staphylococcus aureus*. He received broad-spectrum antimicrobials, wound care, analgesia; serial debridement (fasciotomy, carpal tunnel decompression); tecovirimat 200 mg IV BID plus ART (tenofovir, lamivudine, dolutegravir). Despite treatment, he died (day 30).

### Intrafamilial transmission mapping

The index case resided in Belo Horizonte, Brazil, alongside 13 family members in a shared household that included cats and dogs and was surrounded by a dense area of vegetation. During the home visit, we observed that the patient’s mother (P1) and a female friend (P7) both had skin lesions that appeared to be indicative of mpox. Samples were collected from both individuals for analysis as part of this study, and they were also referred to by local healthcare centers for a clinical evaluation of the lesions. The children, nieces, and nephews of the index case were not present during this initial visit. Afterward, we were informed that they had developed lesions consistent with mpox. The physicians from the study team evaluated their condition, and clinical samples were collected.

**Fig 2:**
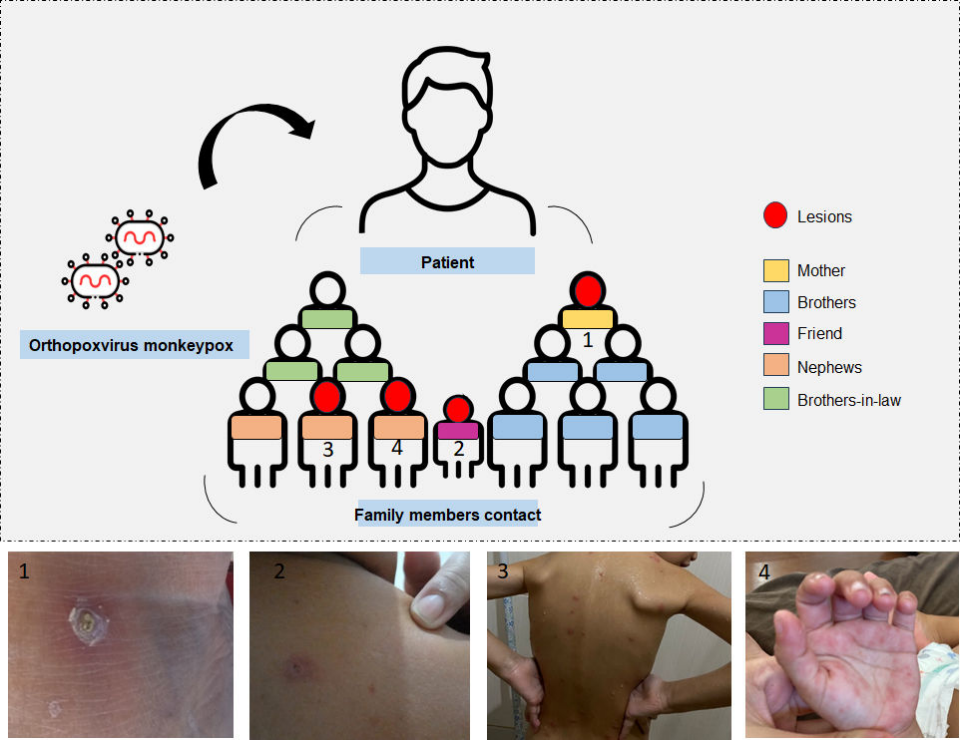
Family cluster and lesion presentation among affected members. Members are labeled P1–P13 and color-coded by relationship to the index case (yellow, mother; blue, siblings; green, siblings-in-law; orange, nieces/nephews; pink, friend). Patients with typical poxvirus lesions are marked with red circles (P1, P7, P11, P12). Representative lesion photographs are shown for P1, P7, P11, and P12.

Although the children had no direct contact with their uncle, who was in isolation, their parents had close contact with him until his death, suggesting possible indirect transmission, either through asymptomatic parents or through contaminated objects. The clinical and demographic data are presented in Table 1, and the family cluster and lesions are illustrated in Fig. 2.

**TABLE 1:** Demographic, clinical, and virological data of the family^*a*^^,^^*b*^^,^^*c*^

Code	Family member	Age/sex	Poxvirus-like lesions/symptoms	Sample type B/OS/P	Viral isolation	Smallpox vaccine	PRNT_50_
P1	Mother	70/F	Poxvirus-like lesion on the foot, cervical lymphadenopathy, malaise, pruritus	Y/Y/Y	–	+	1:40
P2	Sister	29/F	Weakness persisting for 15 days	Y/Y/N	–	–	≤1:10
P3	Sister	40/F	Weakness persisting for 3 days	Y/Y/N	–	–	≤1:10
P4	Sister	35/F	NS	Y/Y/N	–	–	≤1:10
P5	Sister	39/F	NS	Y/Y/N	–	–	≤1:10
P6	Brother	37/M	Fever, severe weight loss, cervical lymphadenopathy, arthralgia, choluria	Y/Y/N	–	–	≤1:10
P7	Female friend	34/F	Poxvirus-like lesion on the back, myalgia, severe weight loss, headache, diarrhea, choluria, retroorbital pain, pharyngeal swelling, chills	Y/Y/Y	–	–	≤1:10
P8	Brother-in-law	45/M	NS	Y/Y/N	–	–	≤1:10
P9	Sister-in-law	35/F	Myalgia, choluria	Y/Y/N	–	–	≤1:10
P10	Brother-in-law	32/M	NS	Y/Y/N	–	–	≤1:10
P11	Niece	3/F	Poxvirus-like lesion across the entire body, lesions in the oropharynx, fever	Y/Y/Y	+	–	≤1:10
P12	Nephew	6/M	Poxvirus-like lesion across the entire body, lesions in the oropharynx, fever	Y/Y/Y	–	–	≤1:10
P13	Niece	17/F	NS	Y/Y/N	–	–	≤1:10

^*a*^ B, blood; OS, oral swab; P, pustule swab.

^*b*^ NS, no symptoms; +, positive; –, negative.

^*c*^ P1-P13, patient codes.

The family cluster members had a mean age of 32.5 years, ranging from 3 to 70 years, and the majority were female (69.2%). Most individuals reported contact with the index case, except for the two children, as mentioned above. The type of contact varied, including sharing personal hygiene products and physical interactions of unspecified duration.

Among the 13 individuals, only 5 reported no clinical signs or symptoms following exposure to the index case. Among symptomatic individuals, poxvirus-like lesions were identified in four individuals. Clinical manifestations included cervical lymphadenopathy, myalgia, weight loss, and arthralgia, as detailed in Table 1.

### Viral isolation

**Fig 3:**
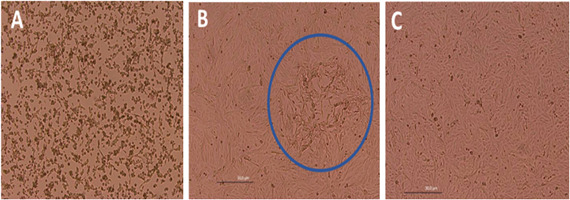
Vero cells inoculated with nasal scab and abdominal pustule material from the index case. (**A and B**) Cytopathic effect with cell rounding, aggregation, cell death, and plaque formation; blue circle highlights a plaque. (**C**) Mock-infected control. Light microscopy, 100×.

Two clinical samples from the index case (day 7; Fig. 1, panels B1 and B4) and from family member P11 (Fig. 2) were positive by viral isolation and qPCR, identifying MPV Clade II (Table S3; Ct 12.3–18.1). MPV grew in Vero cells (Fig. 3; Fig. S1) from the index patient’s nasal scab (Fig. 1, panel B1) and abdominal pustule (Fig. 1, panel B4) and from P11’s scab (Fig. 2), with characteristic cytopathic effect (morphologic changes, clustering, cell death, lysis plaques).

### Evaluation of neutralizing antibodies in family cluster members

**Fig 4:**
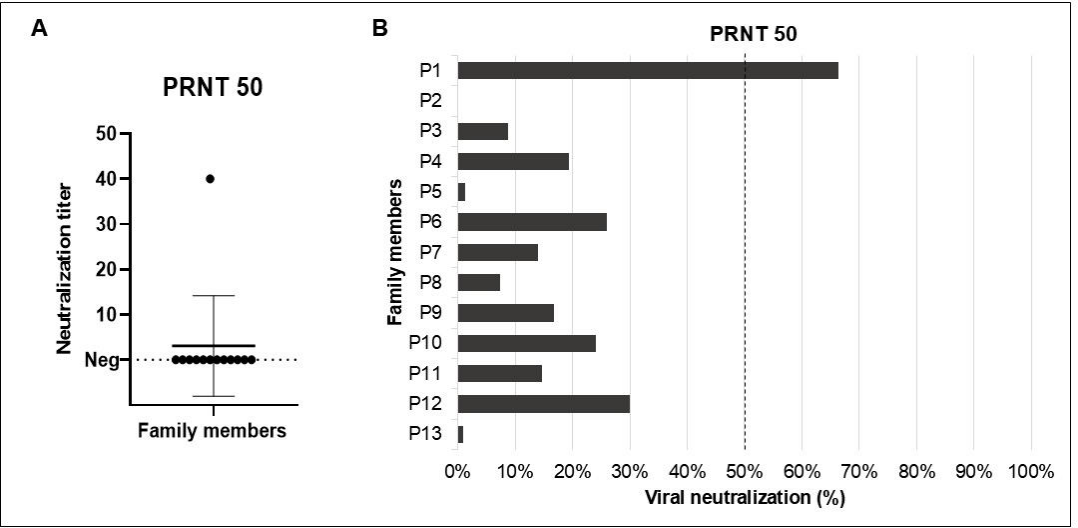
Viral neutralization in sera from family-cluster members. (**A**) Neutralization titers for samples achieving ≥50% viral neutralization; mean ± SD shown. (**B**) Percent neutralization at a 1:10 serum dilution for all tested members.

Using the serum neutralization assay, it was possible to detect the presence of antibodies against OPV with a PRNT_50_ in one family member. The viral neutralization titer obtained was 1:40. However, it is important to consider the history of smallpox vaccination since the only positive sample in the neutralization assay was from a 70-year-old woman (P1) (Fig. 4A). It is also important to note that samples were collected on a day when, except for patients P4, P5, P8, P10, and P13, the other patients exhibited symptoms and lesions. This suggests that they may have been in the acute phase of infection during which serological tests might still return negative results.

Regarding other contacts, the percentage of viral neutralization for each sample at a 1:10 dilution, the lowest dilution tested for all human contacts, was calculated. Through this analysis, it was observed that among the positive samples, one (P12) that was closest to the 50% cutoff, reaching 30% viral neutralization. However, for the other samples, neutralization percentages below 30% were found (Fig. 4B).

### Genomic data corroborates clinical evidence of linked MPV infections

**Fig 5:**
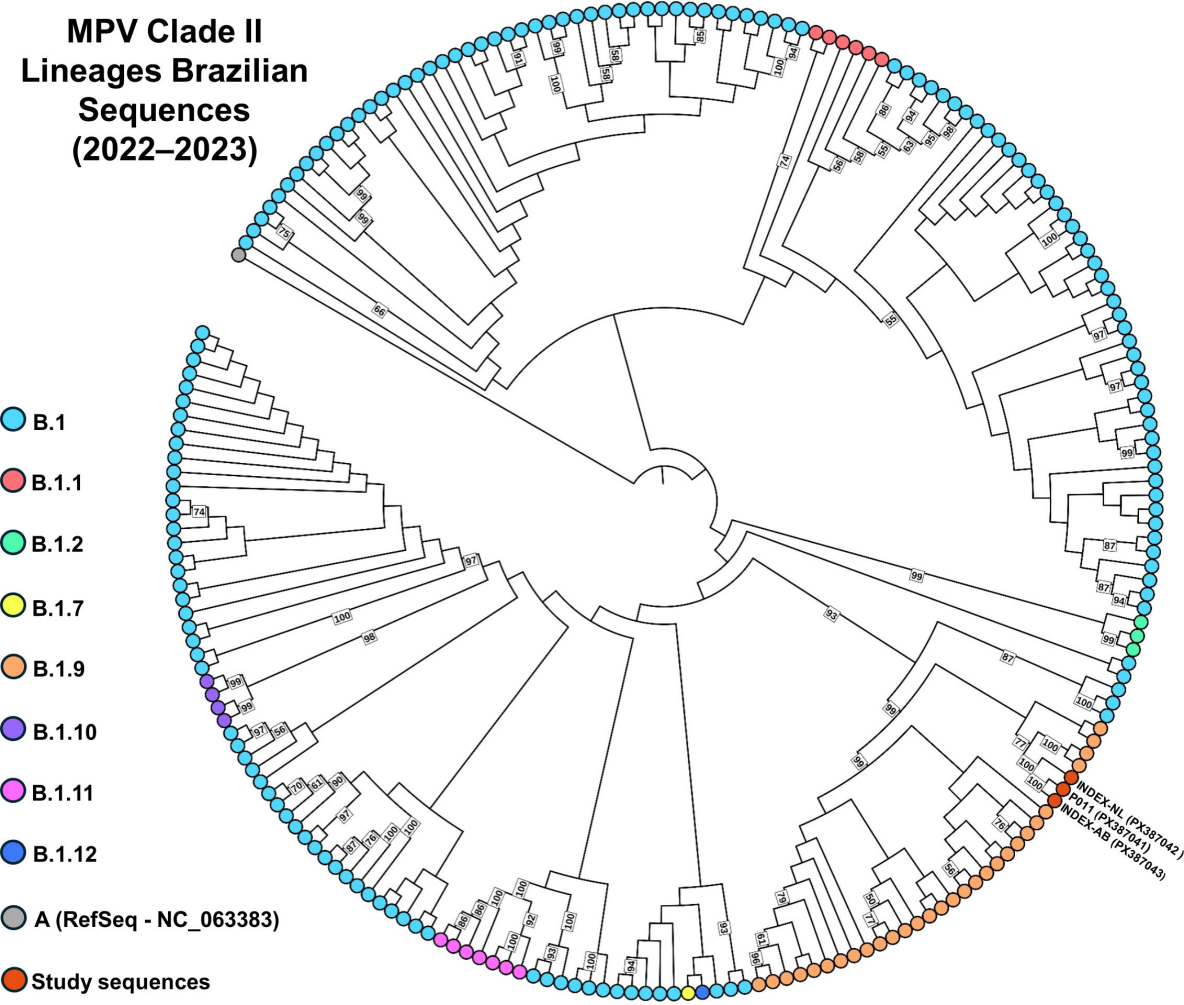
Phylogeny of Brazilian MPV genomes (2022–2023) with lineage assignment of study sequences. Maximum-likelihood tree inferred from 209 Brazilian genomes (194,981 nucleotide sites). Substitution model: TVM+F+I+R3; support from 1,000 bootstrap replicates (node labels shown for values ≥50). Colored circles denote MPV lineages: dark gray, A; light cyan, B.1; red, B.1.1; light green, B.1.2; yellow, B.1.7; light orange, B.1.9; light purple, B.1.10; pink, B.1.11; dark blue, B.1.12; dark orange, sequences generated in this study PO11 (PX387041), INDEX-NL (PX387042), and INDEX-AB (PX387043).

**Fig 6:**
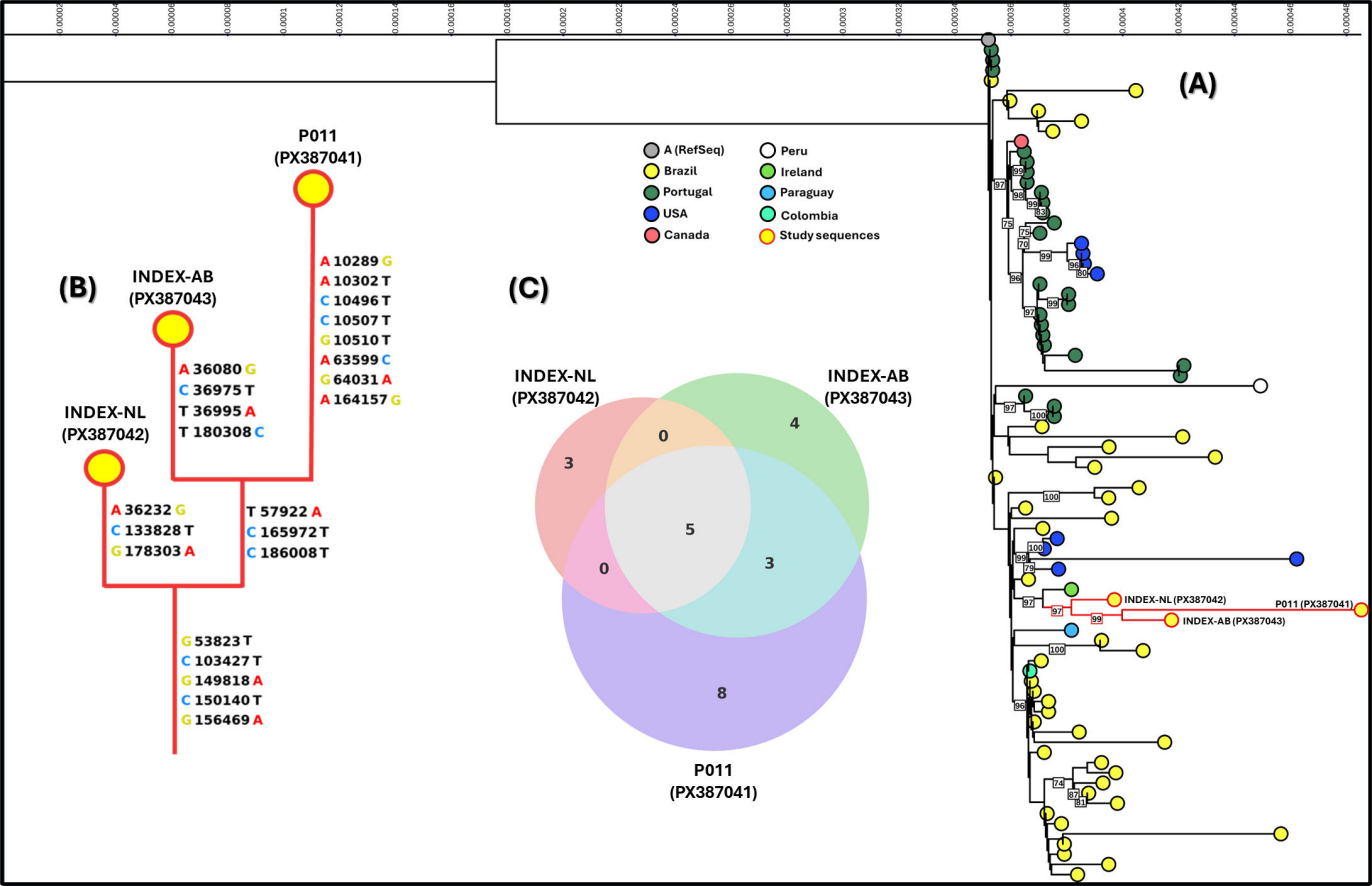
Phylogeny and mutations within MPV lineage B.1.9. (**A**) Maximum-likelihood tree from 83 lineage B.1.9 genomes (195,020 aligned nucleotide sites). Substitution model: HKY+F+I; support from 1,000 bootstrap replicates (node labels shown for values ≥70). Colored circles indicate sampling locations: dark gray, RefSeq; yellow, Brazil; dark green, Portugal and Ireland; dark blue, USA; light red, Canada; white, Peru; blue, Paraguay; cyan, Colombia; study sequences highlighted in red, PO11 (PX387041), INDEX-NL (PX387042), and INDEX-AB (PX387043). (**B**) Consensus cladogram represents the most likely hypothesis for the clade formed by the three study sequences. Mutations are mapped onto the branches where they are most likely to have occurred. The letter on the left and the central number refer to the base and its position in the reference sequence, while the letter on the right represents the corresponding base in the study sequence. (**C**) Venn diagram showing shared and unique mutations among the three study sequences.

We sequenced three viral isolates from the index case’s abdominal pustule and nasal scab, and P11 from a 3-year-old child. All yielded >99% identity and >96% genome coverage vs the MPV Clade II reference (GenBank: NC_063383), with mean depth >500× (Table S4). To test the clinically suggested common source, sequences were first genotyped with Nextclade v3.15.3, and all three sequences were classified as Clade IIb, lineage B.1.9. A phylogeny including these sequences and 209 Brazilian MPV genomes from 2022 to 2023 (Fig. 5) confirmed this, clustering the three within a B.1.9 clade (bootstrap 99). The outbreak samples formed a single well-supported clade, and the index case’s abdominal pustule and the P11-niece sequence were closest. To refine relationships within B.1.9, we built a lineage-restricted tree using the three targets plus 80 sequences (Fig. 6A). Results matched the initial phylogeny, and the three outbreak genomes formed one strongly supported clade, with the abdominal pustule and P11-niece most closely related. This clade grouped with an Irish sample, within a broader clade dominated by Brazilian genomes.

Compared with the B.1.9 “founder” (EPI_ISL_13056892, https://clades.nextstrain.org), the index case’s nasal scab, abdominal pustule, and P11-niece sequences carried 8, 12, and 16 unique mutations, respectively. All three shared five mutations; the abdominal lesion and P11-niece shared three additional ones, and the remainder were unique to each (Fig. 6B through C; Table S5). These patterns align with the clinical timeline, in which the nasal scab at day 7 (Fig. 1, panel B) preceded the abdominal pustule (Fig. 1, panel B4) and the niece’s symptoms.

## DISCUSSION

In contrast to earlier outbreaks which were largely zoonotic and confined to endemic regions of Africa, the 2022 mpox outbreak was characterized by sustained human-to-human transmission in non-endemic countries. Many cases occurred without a history of travel or animal exposure, and the transmission was predominantly associated with sexual contact, particularly among men who have sex with men (26). Clinically, recent cases showed shorter incubation, fewer systemic symptoms, and localized genital/perianal lesions vs earlier widespread rash and more severe systemic illness (27). Mpox should be included in the differential diagnosis of vesiculopustular rashes (herpes simplex virus, syphilis, varicella, molluscum contagiosum, rickettsioses, measles), and isolation precautions should be maintained until mpox is ruled out (26). This is pertinent here, as appropriate isolation could have reduced domestic spread. Intrafamilial transmission can occur via non-sexual close contact, contaminated materials (bedding/dressings), or respiratory droplets during prolonged exposure (28). We report a fatal MPV infection in a patient with active HIV and severe CD4^+^ depletion, syphilis, and nosocomial MRSA (Fig. 1, panel A). Severe disease aligns with evidence linking profound HIV immunosuppression to poor MPV outcomes (29, 30). Clinical, molecular, and serologic data support the intrafamilial transmission, including persons outside classical risk groups (older women and children; Fig. 2, Table 1), consistent with reports of household spread to women, children, and older adults (29, 31).

In serology, only a 70-year-old woman had OPV-neutralizing antibodies above the PRNT cutoff (Fig. 4), likely reflecting residual immunity from smallpox vaccination, discontinued in Brazil since the late 1970s (32). Others, including children, were negative (Fig. 4). The index patient’s 6-year-old nephew (P12) reached 30% neutralization and had systemic symptoms (Fig. 4B). Some participants were still symptomatic at sampling, which may explain absent antibodies due to early infection. IgM typically appears ~day 5 after rash and the IgG becomes ELISA-detectable from day 8, as in the 2003 U.S. outbreak (33). During the ongoing Clade IIb outbreak, the IgM levels typically rise around 2 weeks after infection (34, 35). In contrast, IgG was detectable in week 1 and continues increasing, peaking ~60 days (34, 35), extending the window for neutralization positivity.

This outbreak involved MPV lineage B.1.9, which reached 93% dominance among sequenced cases in Portugal (36) and, as demonstrated by Godinho et al. (2024), was associated with sustained transmission in Brazil, particularly in Rio Grande do Sul (~60% of genomes) (37). B.1.9 was also detected in Minas Gerais (18). Phylogeny clustered the three genomes from this study on a single branch with maximal support (bootstrap = 100) among Brazilian and international B.1.9 sequences (Fig. 5 and 6), indicating a shared transmission chain.

Across the three genomes, we observed 23 site-specific mutations relative to the Nextclade B.1.9 founder (EPI_ISL_13056892), five shared by all. The abdominal pustule from the index patient and the niece’s sample shared three additional mutations absent in the nasal lesion, suggesting temporal divergence across anatomical sites. In advanced HIV, prolonged MPV replication can drive persistent shedding, higher viral loads, and site-specific mutations, including potential drug-resistance changes, fostering new lineages (38, 39). The within-host evolution has been estimated at ~0.68 SNPs per 3 weeks (40).

One plausible contributor to mutation accumulation in this proofreading DNA virus is host APOBEC3 activity, which introduces characteristic C→T substitutions (41). Here, 12 mutations (~52%) were compatible with APOBEC3 activity. Finally, although the observed punctual genetic differences are compatible with microevolution and short-interval transmission, it is important to acknowledge that, as in any sequencing-based approach, technical limitations may exist.

This MPV infection in an immunocompromised patient reinforces the association between advanced HIV and severe disease. The combined clinical, molecular, serologic, and genomic findings are consistent with intrafamilial transmission. Genomics analysis confirmed B.1.9 infection and revealed intra-host microevolution, including changes consistent with APOBEC3 activity. Our findings suggest that closer genomic and epidemiologic monitoring is warranted, especially in immunocompromised patients where infections may persist and transmission can go unnoticed.

### Article summary

Fatal mpox in an immunocompromised patient and intrafamilial spread to a child underscores the need for strengthened household prevention and genomic–epidemiologic surveillance.

## Data Availability

The authors affirm that all data supporting the findings in this study are accessible in the article and appendix. Source data are provided with this article. The consensus sequences generated in this study have been deposited in GenBank under accession numbers PX387041–PX387043.

## SUPPLEMENTAL MATERIAL

Supplemental tables and figure spectrum.03701-25-s0001.pdf Tables S1 to S5 and Figure S1.

ASM does not own the copyrights to Supplemental Material that may be linked to, or accessed through, an article. The authors have granted ASM a non-exclusive, world-wide license to publish the Supplemental Material files. Please contact the corresponding author directly for reuse.
