# Supplementary material for: Genomic, virologic, and epidemiologic surveillance to track intrafamilial Mpox, Brazil
Source: Microbiol Spectr. 2026 Apr 22;14(6):e03701-25. doi: 10.1128/spectrum.03701-25 (PMC13227957; doi:10.1128/spectrum.03701-25)
Supplement: Supplemental tables and figure — Tables S1 to S5 and Figure S1. [file spectrum.03701-25-s0001.pdf]

SUPPLEMENTAL MATERIAL

Table S1 - Dataset of MPV clade IIb 209 sequences for phylogenetic analysis—Brazil (2022–2023) and lineage genotyping.

Sample name	Accession code	Collection date	Lineage
MPXV-M5312_HM12_RIVERS(REFSEQ)	NC_063383 *	2018-08	A
HMPXV/BRAZIL/RS-IAL-02/2022	EPI_ISL_13234112	2022-05-31	B.1
HMPXV/BRAZIL/RS-IAL-05/2022	EPI_ISL_13343697	2022-06-07	B.1
HMPXV/BRAZIL/SP-IAL-03/2022	EPI_ISL_13314740	2022-06-11	B.1
HMPXV/BRAZIL/SP-IAL-04/2022	EPI_ISL_13343634	2022-06-13	B.1
HMPXV/BRAZIL/SP-IAL-08/2022	EPI_ISL_13436792	2022-06-13	B.1.10
HMPXV/BRAZIL/SP-IAL-07/2022	EPI_ISL_13436658	2022-06-14	B.1.12
HMPXV/BRAZIL/SP-IAL-06/2022	EPI_ISL_13343718	2022-06-15	B.1.10
HMPXV/BRAZIL/RJ-FIOCRUZ-14992/2022	EPI_ISL_13484458	2022-06-16	B.1
HMPXV/BRAZIL/SP-IAL-09/2022	EPI_ISL_13437056	2022-06-20	B.1
HMPXV/BRAZIL/SP-IAL-10/2022	EPI_ISL_13459346	2022-06-21	B.1
HMPXV/BRAZIL/SP-IAL-11/2022	EPI_ISL_13459347	2022-06-22	B.1
HMPXV/BRAZIL/SP-IAL-12/2022	EPI_ISL_13459482	2022-06-22	B.1
HMPXV/BRAZIL/SP-IAL-13/2022	EPI_ISL_13459483	2022-06-23	B.1
HMPXV/BRAZIL/SP-IAL-15/2022	EPI_ISL_13508471	2022-06-24	B.1
HMPXV/BRAZIL/SP-IAL-14/2022	EPI_ISL_13508393	2022-06-24	B.1.7
HMPXV/BRAZIL/MG-FUNED-311261273/2022	EPI_ISL_16650225	2022-07-01	B.1
HMPXV/BRAZIL/MG-FUNED-311261010/2022	EPI_ISL_16650224	2022-07-01	B.1
HMPXV/BRAZIL/SP-IAL-16/2022	EPI_ISL_13705358	2022-07-04	B.1

Sample name	Accession code	Collection date	Lineage
**HMPXV/BRAZIL/MG-FUNED-311261816/2022**	**EPI_ISL_16650230**	**2022-07-04**	**B.1**
**HMPXV/BRAZIL/MG-FUNED-311262116/2022**	**EPI_ISL_16650231**	**2022-07-04**	**B.1**
**HMPXV/BRAZIL/MG-FUNED-311262265/2022**	**EPI_ISL_16650227**	**2022-07-04**	**B.1.1**
**HMPXV/BRAZIL/MG-FUNED-311261841/2022**	**EPI_ISL_16650229**	**2022-07-04**	**B.1**
**HMPXV/BRAZIL/MG-FUNED-311262133/2022**	**EPI_ISL_16650228**	**2022-07-04**	**B.1**
**HMPXV/BRAZIL/MG-FUNED-311265338/2022**	**EPI_ISL_16650238**	**2022-07-05**	**B.1**
**HMPXV/BRAZIL/MG-FUNED-311262687/2022**	**EPI_ISL_16650233**	**2022-07-05**	**B.1**
**HMPXV/BRAZIL/MG-FUNED-311262723/2022**	**EPI_ISL_16650234**	**2022-07-05**	**B.1**
**HMPXV/BRAZIL/SP-IAL-18/2022**	**EPI_ISL_13732932**	**2022-07-06**	**B.1**
**HMPXV/BRAZIL/MG-FUNED-311263902/2022**	**EPI_ISL_16650235**	**2022-07-06**	**B.1**
**HMPXV/BRAZIL/MG-FUNED-311264859/2022**	**EPI_ISL_16650237**	**2022-07-07**	**B.1**
**HMPXV/BRAZIL/MG-FUNED-311266186/2022**	**EPI_ISL_16650240**	**2022-07-08**	**B.1**
**HMPXV/BRAZIL/MG-FUNED-311266133/2022**	**EPI_ISL_16650239**	**2022-07-08**	**B.1**
**HMPXV/BRAZIL/MG-FUNED-311267311/2022**	**EPI_ISL_16650244**	**2022-07-11**	**B.1**
**HMPXV/BRAZIL/MG-FUNED-311267285/2022**	**EPI_ISL_16650242**	**2022-07-11**	**B.1**
**HMPXV/BRAZIL/MG-FUNED-311267938/2022**	**EPI_ISL_16650245**	**2022-07-12**	**B.1**
**HMPXV/BRAZIL/SP-IAL-21/2022**	**EPI_ISL_14021725**	**2022-07-13**	**B.1**
**HMPXV/BRAZIL/MG-FUNED-311271087/2022**	**EPI_ISL_16650246**	**2022-07-15**	**B.1**
**HMPXV/BRAZIL/SP-IAL-25/2022**	**EPI_ISL_14070855**	**2022-07-19**	**B.1**
**HMPXV/BRAZIL/SP-IAL-22/2022**	**EPI_ISL_14070493**	**2022-07-19**	**B.1**
**HMPXV/BRAZIL/SP-IAL-23/2022**	**EPI_ISL_14070852**	**2022-07-19**	**B.1**
**HMPXV/BRAZIL/RJ-FIOCRUZ-29696/2022**	**EPI_ISL_17048208**	**2022-07-21**	**B.1.10**

Sample name	Accession code	Collection date	Lineage
HMPXV/BRAZIL/RS-IAL-40/2022	EPI_ISL_14622913	2022-07-26	B.1.1
HMPXV/BRAZIL/AM-FIOCRUZ-ILMD2204652/2022	EPI_ISL_14467428	2022-07-28	B.1
HMPXV/BRAZIL/RS-IAL-41/2022	EPI_ISL_14622953	2022-07-29	B.1
HMPXV/BRAZIL/RJ-LVM_0451-4/2022	EPI_ISL_18241788	2022-08-01	B.1
HMPXV/BRAZIL/RS-CEVS-CDCT45/2022	EPI_ISL_15165614	2022-08-01	B.1
HMPXV/BRAZIL/PR-IAL-26/2022	EPI_ISL_14414948	2022-08-01	B.1
HMPXV/BRAZIL/SP-IAL-36/2022	EPI_ISL_14622520	2022-08-02	B.1
HMPXV/BRAZIL/BA-FIOCRUZ-29688/2022	EPI_ISL_17048205	2022-08-02	B.1
HMPXV/BRAZIL/BA-FIOCRUZ-29694/2022	EPI_ISL_17048207	2022-08-02	B.1
HMPXV/BRAZIL/BA-FIOCRUZ-29690/2022	EPI_ISL_17048206	2022-08-02	B.1
HMPXV/BRAZIL/BA-FIOCRUZ-29681/2022	EPI_ISL_17048204	2022-08-02	B.1
HMPXV/BRAZIL/SP-IAL-43/2022	EPI_ISL_14623175	2022-08-03	B.1
HMPXV/BRAZIL/SP-IAL-35/2022	EPI_ISL_14622055	2022-08-03	B.1.1
HMPXV/BRAZIL/SP-IAL-47/2022	EPI_ISL_14624610	2022-08-03	B.1
HMPXV/BRAZIL/SP-IAL-46/2022	EPI_ISL_14624411	2022-08-03	B.1
HMPXV/BRAZIL/SP-IAL-48/2022	EPI_ISL_14624698	2022-08-03	B.1
HMPXV/BRAZIL/SP-IAL-45/2022	EPI_ISL_14623704	2022-08-04	B.1
HMPXV/BRAZIL/SP-IAL-38/2022	EPI_ISL_14622706	2022-08-04	B.1.1
HMPXV/BRAZIL/SP-IAL-39/2022	EPI_ISL_14622707	2022-08-04	B.1
HMPXV/BRAZIL/SP-IAL-37/2022	EPI_ISL_14622705	2022-08-04	B.1
HMPXV/BRAZIL/SP-IAL-51/2022	EPI_ISL_14625156	2022-08-04	B.1
HMPXV/BRAZIL/RS-CEVS-CDCT71/2022	EPI_ISL_15165618	2022-08-04	B.1.9

Sample name	Accession code	Collection date	Lineage
HMPXV/BRAZIL/RS-CEVS-CDCT60/2022	EPI_ISL_14465517	2022-08-04	B.1
HMPXV/BRAZIL/RS-CEVS-CDCT66/2022	EPI_ISL_15165616	2022-08-04	B.1.9
HMPXV/BRAZIL/PR-IAL-55/2022	EPI_ISL_14625256	2022-08-05	B.1
HMPXV/BRAZIL/SP-IAL-31/2022	EPI_ISL_14571439	2022-08-05	B.1
HMPXV/BRAZIL/SP-IAL-29/2022	EPI_ISL_14571433	2022-08-05	B.1
HMPXV/BRAZIL/SP-IAL-53/2022	EPI_ISL_14625190	2022-08-05	B.1
HMPXV/BRAZIL/RJ-FIOCRUZ-29778/2022	EPI_ISL_17471103	2022-08-05	B.1.9
HMPXV/BRAZIL/MG-FUNED-311283035/2022	EPI_ISL_16650248	2022-08-05	B.1
HMPXV/BRAZIL/SP-IAL-28/2022	EPI_ISL_14571429	2022-08-07	B.1
HMPXV/BRAZIL/RJ-FIOCRUZ-29779/2022	EPI_ISL_17471102	2022-08-07	B.1
HMPXV/BRAZIL/SP-IAL-52/2022	EPI_ISL_14625157	2022-08-08	B.1.10
HMPXV/BRAZIL/SP-IAL-56/2022	EPI_ISL_14625282	2022-08-08	B.1
HMPXV/BRAZIL/RJ-FIOCRUZ-29782/2022	EPI_ISL_17471104	2022-08-08	B.1
HMPXV/BRAZIL/SP-IAL-82/2022	EPI_ISL_14995586	2022-08-08	B.1
HMPXV/BRAZIL/SP-IAL-32/2022	EPI_ISL_14571441	2022-08-09	B.1
HMPXV/BRAZIL/SP-IAL-33/2022	EPI_ISL_14571442	2022-08-09	B.1
HMPXV/BRAZIL/SP-IAL-34/2022	EPI_ISL_14571444	2022-08-09	B.1.1
HMPXV/BRAZIL/SP-IAL-30/2022	EPI_ISL_14571435	2022-08-09	B.1
HMPXV/BRAZIL/RJ-FIOCRUZ-29780/2022	EPI_ISL_17471100	2022-08-10	B.1
HMPXV/BRAZIL/RS-CEVS-CDCT131/2022	EPI_ISL_15165603	2022-08-10	B.1.9
HMPXV/BRAZIL/RS-CEVS-CDCT148/2022	EPI_ISL_15165604	2022-08-10	B.1.9
HMPXV/BRAZIL/RS-CEVS-CDCT184/2022	EPI_ISL_15165608	2022-08-11	B.1.9

Sample name	Accession code	Collection date	Lineage
HMPXV/BRAZIL/MG-FUNED-311287351/2022	EPI_ISL_16650247	2022-08-12	B.1
HMPXV/BRAZIL/RS-CEVS-CDCT239/2022	EPI_ISL_17406096	2022-08-15	B.1.9
HMPXV/BRAZIL/MG-FUNED-311288391/2022	EPI_ISL_16650249	2022-08-15	B.1
HMPXV/BRAZIL/RS-CEVS-CDCT200/2022	EPI_ISL_17406095	2022-08-16	B.1.9
HMPXV/BRAZIL/SP-IAL-61/2022	EPI_ISL_14772914	2022-08-17	B.1
HMPXV/BRAZIL/RS-CEVS-CDCT329/2022	EPI_ISL_17406097	2022-08-17	B.1
HMPXV/BRAZIL/SP-IAL-59/2022	EPI_ISL_14772912	2022-08-18	B.1
HMPXV/BRAZIL/RS-CEVS-CDCT357/2022	EPI_ISL_17406098	2022-08-18	B.1.9
HMPXV/BRAZIL/RS-CEVS-CDCT441/2022	EPI_ISL_17406100	2022-08-18	B.1.9
HMPXV/BRAZIL/RS-CEVS-CDCT404/2022	EPI_ISL_17406099	2022-08-18	B.1
HMPXV/BRAZIL/SP-IAL-63/2022	EPI_ISL_14809096	2022-08-19	B.1.1
HMPXV/BRAZIL/PR-IAL-66/2022	EPI_ISL_14809099	2022-08-19	B.1
HMPXV/BRAZIL/SP-IAL-62/2022	EPI_ISL_14773001	2022-08-22	B.1
HMPXV/BRAZIL/SP-IAL-60/2022	EPI_ISL_14772913	2022-08-22	B.1
HMPXV/BRAZIL/MG-FUNED-311291580/2022	EPI_ISL_16650262	2022-08-22	B.1
HMPXV/BRAZIL/SP-IAL-67/2022	EPI_ISL_14809100	2022-08-23	B.1
HMPXV/BRAZIL/RS-CEVS-CDCT599/2022	EPI_ISL_17406101	2022-08-23	B.1
HMPXV/BRAZIL/RS-CEVS-CDCT628/2022	EPI_ISL_17406102	2022-08-23	B.1
HMPXV/BRAZIL/SP-IAL-68/2022	EPI_ISL_14865785	2022-08-24	B.1
HMPXV/BRAZIL/SP-IAL-71/2022	EPI_ISL_14866751	2022-08-24	B.1
HMPXV/BRAZIL/RS-CEVS-CDCT733/2022	EPI_ISL_17406103	2022-08-24	B.1.9
HMPXV/BRAZIL/SP-IAL-69/2022	EPI_ISL_14866481	2022-08-25	B.1

Sample name	Accession code	Collection date	Lineage
HMPXV/BRAZIL/SP-IAL-72/2022	EPI_ISL_14866752	2022-08-25	B.1
HMPXV/BRAZIL/PI-IAL-81/2022	EPI_ISL_14995585	2022-08-25	B.1
HMPXV/BRAZIL/MG-FUNED-311294876/2022	EPI_ISL_16650251	2022-08-26	B.1
HMPXV/BRAZIL/BA-IAL-91/2022	EPI_ISL_14995612	2022-08-26	B.1
HMPXV/BRAZIL/RS-CEVS-CDCT875/2022	EPI_ISL_17406104	2022-08-26	B.1.9
HMPXV/BRAZIL/RS-CEVS-CDCT883/2022	EPI_ISL_17406105	2022-08-26	B.1.9
HMPXV/BRAZIL/SP-IAL-74/2022	EPI_ISL_14995206	2022-08-29	B.1
HMPXV/BRAZIL/RS-CEVS-CDCT925/2022	EPI_ISL_17406106	2022-08-29	B.1.9
HMPXV/BRAZIL/SP-IAL-79/2022	EPI_ISL_14995582	2022-08-29	B.1
HMPXV/BRAZIL/SP-IAL-83/2022	EPI_ISL_14995587	2022-08-30	B.1
HMPXV/BRAZIL/SP-IAL-77/2022	EPI_ISL_14995580	2022-08-30	B.1
HMPXV/BRAZIL/RS-CEVS-CDCT1006/2022	EPI_ISL_17406093	2022-08-30	B.1.9
HMPXV/BRAZIL/RS-CEVS-CDCT1100/2022	EPI_ISL_17406094	2022-08-31	B.1.9
HMPXV/BRAZIL/RS-CEVS-CDCT1294/2022	EPI_ISL_17406113	2022-08-31	B.1.9
HMPXV/BRAZIL/SP-IAL-87/2022	EPI_ISL_14995591	2022-09-01	B.1
HMPXV/BRAZIL/SP-IAL-97/2022	EPI_ISL_14995653	2022-09-01	B.1
HMPXV/BRAZIL/SP-IAL-93/2022	EPI_ISL_14995622	2022-09-01	B.1
HMPXV/BRAZIL/SP-IAL-89/2022	EPI_ISL_14995593	2022-09-01	B.1
HMPXV/BRAZIL/SP-IAL-99/2022	EPI_ISL_14995724	2022-09-01	B.1
HMPXV/BRAZIL/SP-IAL-102/2022	EPI_ISL_15419133	2022-09-02	B.1
HMPXV/BRAZIL/SP-IAL-109/2022	EPI_ISL_15419140	2022-09-02	B.1
HMPXV/BRAZIL/BA-IAL-120/2022	EPI_ISL_15419151	2022-09-03	B.1

Sample name	Accession code	Collection date	Lineage
HMPXV/BRAZIL/RS-CEVS-CDCT1450/2022	EPI_ISL_17406110	2022-09-03	B.1.9
HMPXV/BRAZIL/SP-IAL-98/2022	EPI_ISL_14995723	2022-09-04	B.1
HMPXV/BRAZIL/SP-IAL-105/2022	EPI_ISL_15419136	2022-09-05	B.1
HMPXV/BRAZIL/SP-IAL-104/2022	EPI_ISL_15419135	2022-09-05	B.1
HMPXV/BRAZIL/SP-IAL-108/2022	EPI_ISL_15419139	2022-09-05	B.1
HMPXV/BRAZIL/SP-IAL-106/2022	EPI_ISL_15419137	2022-09-05	B.1
HMPXV/BRAZIL/SP-IAL-113/2022	EPI_ISL_15419144	2022-09-05	B.1
HMPXV/BRAZIL/RS-CEVS-CDCT1492/2022	EPI_ISL_17406112	2022-09-05	B.1.9
HMPXV/BRAZIL/SP-IAL-112/2022	EPI_ISL_15419143	2022-09-06	B.1
HMPXV/BRAZIL/SP-IAL-103/2022	EPI_ISL_15419134	2022-09-06	B.1
HMPXV/BRAZIL/SP-IAL-107/2022	EPI_ISL_15419138	2022-09-06	B.1
HMPXV/BRAZIL/PI-IAL-127/2022	EPI_ISL_15419158	2022-09-06	B.1
HMPXV/BRAZIL/SP-IAL-100/2022	EPI_ISL_15419131	2022-09-06	B.1
HMPXV/BRAZIL/SP-IAL-110/2022	EPI_ISL_15419141	2022-09-07	B.1
HMPXV/BRAZIL/SP-IAL-111/2022	EPI_ISL_15419142	2022-09-08	B.1
HMPXV/BRAZIL/SP-IAL-114/2022	EPI_ISL_15419145	2022-09-08	B.1
HMPXV/BRAZIL/SP-IAL-117/2022	EPI_ISL_15419148	2022-09-08	B.1
HMPXV/BRAZIL/MG-FUNED-311300630/2022	EPI_ISL_16650258	2022-09-08	B.1
HMPXV/BRAZIL/SP-IAL-123/2022	EPI_ISL_15419154	2022-09-09	B.1
HMPXV/BRAZIL/SP-IAL-115/2022	EPI_ISL_15419146	2022-09-09	B.1
HMPXV/BRAZIL/SP-IAL-116/2022	EPI_ISL_15419147	2022-09-09	B.1
HMPXV/BRAZIL/SP-IAL-128/2022	EPI_ISL_15419159	2022-09-09	B.1

Sample name	Accession code	Collection date	Lineage
**HMPXV/BRAZIL/RS-CEVS-CDCT1667/2022**	**EPI_ISL_17406111**	**2022-09-09**	**B.1.9**
**HMPXV/BRAZIL/SP-IAL-124/2022**	**EPI_ISL_15419155**	**2022-09-10**	**B.1**
**HMPXV/BRAZIL/SP-IAL-122/2022**	**EPI_ISL_15419153**	**2022-09-11**	**B.1**
**HMPXV/BRAZIL/SP-IAL-125/2022**	**EPI_ISL_15419156**	**2022-09-12**	**B.1**
**HMPXV/BRAZIL/SP-IAL-126/2022**	**EPI_ISL_15419157**	**2022-09-12**	**B.1**
**HMPXV/BRAZIL/SP-IAL-121/2022**	**EPI_ISL_15419152**	**2022-09-12**	**B.1**
**HMPXV/BRAZIL/RS-CEVS-CDCT1719/2022**	**EPI_ISL_17406119**	**2022-09-12**	**B.1.9**
**HMPXV/BRAZIL/RS-CEVS-CDCT1661/2022**	**EPI_ISL_17406118**	**2022-09-12**	**B.1.9**
**HMPXV/BRAZIL/MG-FUNED-311303564/2022**	**EPI_ISL_16650260**	**2022-09-13**	**B.1**
**HMPXV/BRAZIL/SP-IAL-131/2022**	**EPI_ISL_15419162**	**2022-09-14**	**B.1**
**HMPXV/BRAZIL/SP-IAL-130/2022**	**EPI_ISL_15419161**	**2022-09-15**	**B.1**
**HMPXV/BRAZIL/RS-CEVS-CDCT2211/2022**	**EPI_ISL_17406115**	**2022-09-25**	**B.1.9**
**HMPXV/BRAZIL/RS-CEVS-CDCT2177/2022**	**EPI_ISL_17406123**	**2022-09-26**	**B.1.9**
**HMPXV/BRAZIL/RS-CEVS-CDCT2200/2022**	**EPI_ISL_17406122**	**2022-09-27**	**B.1.9**
**HMPXV/BRAZIL/RS-CEVS-CDCT2216/2022**	**EPI_ISL_17406120**	**2022-09-27**	**B.1**
**HMPXV/BRAZIL/RS-CEVS-CDCT2230/2022**	**EPI_ISL_17406117**	**2022-09-27**	**B.1**
**HMPXV/BRAZIL/RJ-FIOCRUZ-29786/2022**	**EPI_ISL_17614034**	**2022-09-30**	**B.1**
**HMPXV/BRAZIL/RJ-FIOCRUZ-29787/2022**	**EPI_ISL_17471110**	**2022-09-30**	**B.1**
**HMPXV/BRAZIL/RJ-FIOCRUZ-29784/2022**	**EPI_ISL_17471106**	**2022-09-30**	**B.1**
**HMPXV/BRAZIL/RJ-FIOCRUZ-29792/2022**	**EPI_ISL_17614049**	**2022-10-04**	**B.1**
**HMPXV/BRAZIL/RS-CEVS-CDCT2376/2022**	**EPI_ISL_17406124**	**2022-10-06**	**B.1.9**
**HMPXV/BRAZIL/AM-CEVS-CDCT2384/2022**	**EPI_ISL_17406108**	**2022-10-07**	**B.1.9**

Sample name	Accession code	Collection date	Lineage
HMPXV/BRAZIL/RS-CEVS-CDCT2464/2022	EPI_ISL_17406109	2022-10-11	B.1.9
HMPXV/BRAZIL/RJ-FIOCRUZ-29802/2022	EPI_ISL_17614022	2022-10-18	B.1.9
HMPXV/BRAZIL/RJ-FIOCRUZ-29800/2022	EPI_ISL_17614037	2022-10-18	B.1
HMPXV/BRAZIL/RJ-FIOCRUZ-29655/2022	EPI_ISL_16871158	2022-10-18	B.1
HMPXV/BRAZIL/RJ-FIOCRUZ-29799/2022	EPI_ISL_17614029	2022-10-18	B.1
HMPXV/BRAZIL/RJ-FIOCRUZ-29803/2022	EPI_ISL_17471108	2022-10-21	B.1
HMPXV/BRAZIL/SP-USP-IMT-0711161/2022	EPI_ISL_17703744	2022-10-31	B.1
HMPXV/BRAZIL/SP-USP-IMT-0811161/2022	EPI_ISL_17703746	2022-10-31	B.1
HMPXV/BRAZIL/SP-USP-IMT-0511161/2022	EPI_ISL_17703745	2022-10-31	B.1
HMPXV/BRAZIL/RJ-FIOCRUZ-29805/2022	EPI_ISL_17614043	2022-10-31	B.1
HMPXV/BRAZIL/RJ-FIOCRUZ-29806/2022	EPI_ISL_17614026	2022-11-01	B.1
HMPXV/BRAZIL/RJ-FIOCRUZ-29807/2022	EPI_ISL_17614025	2022-11-04	B.1
HMPXV/BRAZIL/RJ-FIOCRUZ-30463/2022	EPI_ISL_19408592	2022-11-08	B.1
HMPXV/BRAZIL/RJ-FIOCRUZ-29809/2022	EPI_ISL_17614036	2022-11-10	B.1
HMPXV/BRAZIL/RJ-FIOCRUZ-29650/2022	EPI_ISL_16871160	2022-11-10	B.1
HMPXV/BRAZIL/RJ-FIOCRUZ-29811/2022	EPI_ISL_17614046	2022-11-14	B.1
HMPXV/BRAZIL/RJ-FIOCRUZ-29651/2022	EPI_ISL_16871161	2022-11-16	B.1
HMPXV/BRAZIL/RJ-FIOCRUZ-29814/2022	EPI_ISL_17614024	2022-11-24	B.1
HMPXV/BRAZIL/RJ-FIOCRUZ-29653/2022	EPI_ISL_16871162	2022-12-11	B.1
HMPXV/BRAZIL/RJ-FIOCRUZ-29816/2022	EPI_ISL_17614048	2022-12-11	B.1
HMPXV/BRAZIL/RJ-FIOCRUZ-29654/2022	EPI_ISL_16871163	2022-12-22	B.1.2
HMPXV/BRAZIL/RJ-FIOCRUZ-3015/2023	EPI_ISL_17614021	2023-01-26	B.1.2

Sample name	Accession code	Collection date	Lineage
HMPXV/BRAZIL/RJ-FIOCRUZ-3016/2023	EPI_ISL_17614040	2023-02-08	B.1.2
HMPXV/BRAZIL/RJ-FIOCRUZ-3608/2023	EPI_ISL_17536782	2023-03-24	B.1.11
HMPXV/BRAZIL/RJ-FIOCRUZ-3608-1P/2023	EPI_ISL_19205402	2023-03-24	B.1.11
HMPXV/BRAZIL/RJ-FIOCRUZ-3610/2023	EPI_ISL_17536784	2023-03-30	B.1.11
HMPXV/BRAZIL/RJ-FIOCRUZ-3611/2023	EPI_ISL_17536785	2023-04-03	B.1.11
HMPXV/BRAZIL/RJ-FIOCRUZ-3611-1P/2023	EPI_ISL_19205405	2023-04-03	B.1.11
HMPXV/BRAZIL/RJ-FIOCRUZ-4194/2023	EPI_ISL_17614030	2023-04-06	B.1.11
HMPXV/BRAZIL/RJ-FIOCRUZ-4196/2023	EPI_ISL_17614042	2023-04-13	B.1.11
HMPXV/BRAZIL/RS-CEVS-362/2023	EPI_ISL_18971016	2023-09-14	B.1
HMPXV/BRAZIL/SP-IAL-3280/2023	EPI_ISL_19446284	2023-10-09	B.1
HMPXV/BRAZIL/SP-IAL-3281/2023	EPI_ISL_19446285	2023-10-09	B.1
HMPXV/BRAZIL/SP-IAL-3282/2023	EPI_ISL_19446286	2023-10-11	B.1
HMPXV/BRAZIL/SP-IAL-357399029/2023	EPI_ISL_19446287	2023-11-29	B.1
HMPXV/BRAZIL/SP-IAL-3284/2023	EPI_ISL_19446288	2023-12-18	B.1

*The reference sequence accession number is from GenBank, whereas all other accession numbers are from the GISAID database.

Table S2 – Dataset of MPV Clade IIb B.1.9 Lineage with 80 Sequences Used for Phylogenetic Analysis

Sample name	Accession number	Country	Collection date
hMpxV/Brazil/SP-IAL-17/2022	EPI_ISL_13705407	Brazil	2022-07-04
hMpxV/Brazil/RJ-LVM_0085-2/2022	EPI_ISL_18241791	Brazil	2022-07-07
hMpxV/Brazil/MG-FUNED-311266796/2022	EPI_ISL_16650243	Brazil	2022-07-11

Sample name	Accession number	Country	Collection date
hMpxV/Brazil/RJ-LVM_0157-1/2022	EPI_ISL_18241790	Brazil	2022-07-14
hMpxV/Brazil/RJ-LVM_0283/2022	EPI_ISL_18241789	Brazil	2022-07-25
hMpxV/Brazil/SP-IAL-42/2022	EPI_ISL_14622960	Brazil	2022-07-31
hMpxV/Brazil/SP-IAL-44/2022	EPI_ISL_14623523	Brazil	2022-08-03
hMpxV/Brazil/AM-FIOCRUZ-ILMD2204653/2022	EPI_ISL_14467429	Brazil	2022-08-04
hMpxV/Brazil/SP-IAL-49/2022	EPI_ISL_14624832	Brazil	2022-08-04
hMpxV/Brazil/RS-CEVS-CDCT71/2022	EPI_ISL_15165618	Brazil	2022-08-04
hMpxV/Brazil/RS-CEVS-CDCT66/2022	EPI_ISL_15165616	Brazil	2022-08-04
hMpxV/Brazil/RJ-FIOCRUZ-29778/2022	EPI_ISL_17471103	Brazil	2022-08-05
hMpxV/Brazil/RJ-FIOCRUZ-29783/2022	EPI_ISL_17471101	Brazil	2022-08-07
hMpxV/Brazil/SP-IAL-82/2022	EPI_ISL_14995586	Brazil	2022-08-08
hMpxV/Brazil/RS-CEVS-CDCT131/2022	EPI_ISL_15165603	Brazil	2022-08-10
hMpxV/Brazil/RS-CEVS-CDCT148/2022	EPI_ISL_15165604	Brazil	2022-08-10
hMpxV/Brazil/RS-CEVS-CDCT184/2022	EPI_ISL_15165608	Brazil	2022-08-11
hMpxV/Brazil/RS-CEVS-CDCT185/2022	EPI_ISL_15165609	Brazil	2022-08-11
hMpxV/Brazil/RS-CEVS-CDCT183/2022	EPI_ISL_15165607	Brazil	2022-08-12
hMpxV/Brazil/RS-CEVS-CDCT239/2022	EPI_ISL_17406096	Brazil	2022-08-15
hMpxV/Brazil/RS-CEVS-CDCT200/2022	EPI_ISL_17406095	Brazil	2022-08-16
hMpxV/Brazil/RS-CEVS-CDCT357/2022	EPI_ISL_17406098	Brazil	2022-08-18
hMpxV/Brazil/RS-CEVS-CDCT441/2022	EPI_ISL_17406100	Brazil	2022-08-18
hMpxV/Brazil/SP-IAL-65/2022	EPI_ISL_14809098	Brazil	2022-08-24
hMpxV/Brazil/RS-CEVS-CDCT733/2022	EPI_ISL_17406103	Brazil	2022-08-24

Sample name	Accession number	Country	Collection date
hMpxV/Brazil/RS-CEVS-CDCT875/2022	EPI_ISL_17406104	Brazil	2022-08-26
hMpxV/Brazil/RS-CEVS-CDCT1006/2022	EPI_ISL_17406093	Brazil	2022-08-30
hMpxV/Brazil/RS-CEVS-CDCT1294/2022	EPI_ISL_17406113	Brazil	2022-08-31
hMpxV/Brazil/BA-IAL-92/2022	EPI_ISL_14995619	Brazil	2022-08-31
hMpxV/Brazil/RS-CEVS-CDCT1100/2022	EPI_ISL_17406094	Brazil	2022-08-31
hMpxV/Brazil/RS-CEVS-CDCT1450/2022	EPI_ISL_17406110	Brazil	2022-09-03
hMpxV/Brazil/RS-CEVS-CDCT1492/2022	EPI_ISL_17406112	Brazil	2022-09-05
hMpxV/Brazil/RS-CEVS-CDCT1667/2022	EPI_ISL_17406111	Brazil	2022-09-09
hMpxV/Brazil/RS-CEVS-CDCT1719/2022	EPI_ISL_17406119	Brazil	2022-09-12
hMpxV/Brazil/RS-CEVS-CDCT2177/2022	EPI_ISL_17406123	Brazil	2022-09-26
hMpxV/Brazil/RS-CEVS-CDCT2200/2022	EPI_ISL_17406122	Brazil	2022-09-27
hMpxV/Brazil/RJ-FIOCRUZ-29794/2022	EPI_ISL_17614023	Brazil	2022-10-06
hMpxV/Brazil/AM-CEVS-CDCT2384/2022	EPI_ISL_17406108	Brazil	2022-10-07
hMpxV/Brazil/RJ-FIOCRUZ-29802/2022	EPI_ISL_17614022	Brazil	2022-10-18
hMpxV/Brazil/RJ-FIOCRUZ-29820/2022	EPI_ISL_17614032	Brazil	2022-12-27
hMpxV/Brazil/RJ-FIOCRUZ-3014/2023	EPI_ISL_17485343	Brazil	2023-01-02
hMpxV/Canada/un-NML-3336/2022	EPI_ISL_13544256	Canada	2022-06-01
hMpxV/Colombia/DC-INS-0718/2022	EPI_ISL_15455913	Colombia	2022-08-16
hMpxV/Ireland/D-NVRL-Z22IRL00507/2022	EPI_ISL_16510131	Ireland	2022-08-22
MPXV-M5312_HM12_Rivers(RefSeq)	NC_063383*	Nigeria	2018-08
hMpxV/Paraguay/726874/2022	EPI_ISL_17988362	Paraguay	2022-08-24
hMpxV/Peru/LIM-INS-504/2023	EPI_ISL_19343534	Peru	2023-11-02

Sample name	Accession number	Country	Collection date
**hMpxV/Portugal/INSA-PT0008/2022**	**EPI_ISL_13052273**	**Portugal**	**2022-05-15**
**hMpxV/Portugal/INSA-PT0020/2022**	**EPI_ISL_13056897**	**Portugal**	**2022-05-19**
**hMpxV/Portugal/INSA-PT0331/2022**	**EPI_ISL_14934575**	**Portugal**	**2022-05-28**
**hMpxV/Portugal/INSA-PT0039/2022**	**EPI_ISL_13466462**	**Portugal**	**2022-06-02**
**hMpxV/Portugal/INSA-PT0321/2022**	**EPI_ISL_14934566**	**Portugal**	**2022-06-04**
**hMpxV/Portugal/INSA-PT0311/2022**	**EPI_ISL_14934558**	**Portugal**	**2022-06-06**
**hMpxV/Portugal/INSA-PT0045/2022**	**EPI_ISL_13466464**	**Portugal**	**2022-06-06**
**hMpxV/Portugal/INSA-PT0255/2022**	**EPI_ISL_14934509**	**Portugal**	**2022-06-18**
**hMpxV/Portugal/INSA-PT0236/2022**	**EPI_ISL_14752251**	**Portugal**	**2022-06-22**
**hMpxV/Portugal/INSA-PT0215/2022**	**EPI_ISL_14752219**	**Portugal**	**2022-06-24**
**hMpxV/Portugal/INSA-PT0183/2022**	**EPI_ISL_14752177**	**Portugal**	**2022-06-28**
**hMpxV/Portugal/INSA-PT0192/2022**	**EPI_ISL_14752191**	**Portugal**	**2022-06-29**
**hMpxV/Portugal/INSA-PT0178/2022**	**EPI_ISL_14752167**	**Portugal**	**2022-06-30**
**hMpxV/Portugal/INSA-PT0156/2022**	**EPI_ISL_14752131**	**Portugal**	**2022-07-04**
**hMpxV/Portugal/INSA-PT0144/2022**	**EPI_ISL_14752117**	**Portugal**	**2022-07-07**
**hMpxV/Portugal/INSA-PT0381/2022**	**EPI_ISL_15199737**	**Portugal**	**2022-07-11**
**hMpxV/Portugal/INSA-PT0389/2022**	**EPI_ISL_15199745**	**Portugal**	**2022-07-12**
**hMpxV/Portugal/INSA-PT0413/2022**	**EPI_ISL_15199766**	**Portugal**	**2022-07-26**
**hMpxV/Portugal/INSA-0052/2022**	**EPI_ISL_14515176**	**Portugal**	**2022-07-30**
**hMpxV/Portugal/INSA-PT0451/2022**	**EPI_ISL_15199792**	**Portugal**	**2022-08-04**
**hMpxV/Portugal/INSA-PT0456/2022**	**EPI_ISL_15199797**	**Portugal**	**2022-08-04**
**hMpxV/Portugal/INSA-PT0470/2022**	**EPI_ISL_15199863**	**Portugal**	**2022-08-08**

Sample name	Accession number	Country	Collection date
hMpxV/Portugal/INSA-PT0489/2022	EPI_ISL_15199627	Portugal	2022-08-11
hMpxV/Portugal/INSA-PT0510/2022	EPI_ISL_15199644	Portugal	2022-08-12
hMpxV/Portugal/INSA-PT0561/2022	EPI_ISL_15199694	Portugal	2022-08-31
hMpxV/USA/NY-NYCPHL-000158/2022	EPI_ISL_16955295	USA	2022-06-29
hMpxV/USA/NY-NYCPHL-000197/2022	EPI_ISL_17086179	USA	2022-07-01
hMpxV/USA/NY-NYCPHL-000198/2022	EPI_ISL_17104265	USA	2022-07-01
hMpxV/USA/NY-NYCPHL-001101/2022	EPI_ISL_17104619	USA	2022-07-05
hMpxV/USA/NY-NYCPHL-000869/2022	EPI_ISL_17086027	USA	2022-07-07
hMpxV/USA/NY-NYCPHL-000330/2022	EPI_ISL_17086232	USA	2022-08-08
hMpxV/USA/NY-NYCPHL-000422/2022	EPI_ISL_16955316	USA	2022-09-07
hMpxV/USA/NJ-CDC-0005/2022	EPI_ISL_15455863	USA	2022-08

***The reference sequence accession number is from GenBank, whereas all other accession numbers are from the GISAID database.**

Table S3: qPCR data of positive clinical samples for MPV isolation.

Patient	Samples	qPCR E9L	Ct*	qPCR Clade II (rTNF)	Ct*
Index Case	Nasal scab	+	12.7	+	14.9
Index Case	Abdominal pustule	+	13.3	+	16.4
P11	Scab	+	17.2	+	18.1

*(Ct) Cycle threshold

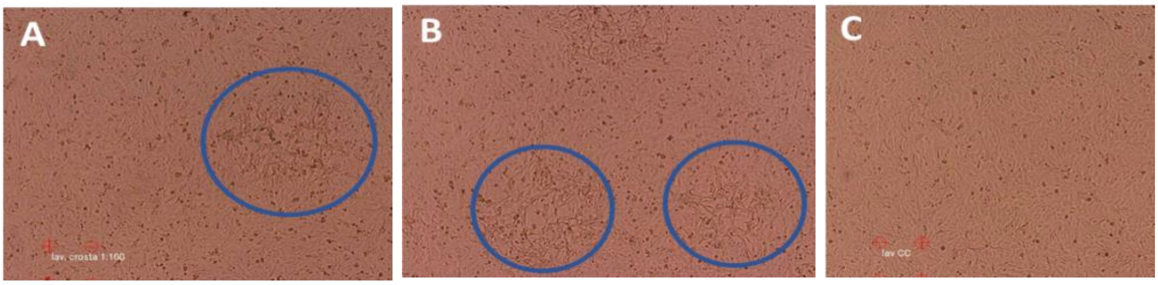

Figure S1. Vero cells inoculated with scab material from patient P11. (A, B) Cytopathic effects, including cell rounding, clustering, cell death, and lytic plaque formation (blue circles). (C) Mock-infected control. Light microscopy; original magnification ×100.

Table S4 - Genome assembly statistics for MPV samples

Sample ID	Number of reads	Genome coverage (%)	Identity Refseq nc_063383 (%)	Average depth (x)	Final genome size (bp)	Accession
**INDEX-NL**	673,87	98.5	99.6	541	197,104	PX387042
**INDEX-AB**	1,722,590	99.0	99.6	1,368	197,173	PX387043
**P011**	903,756	96.2	99.6	714	197,105	PX387041

Table S5 – Mutations in the studied sequences relative to the lineage founder (EPI_ISL_13056892, Nextclade)

Mutation	Position	From	To	Orf	Product*	Amino acid substitutions	Samples with the mutation
1	10289	A	G	OPG021	Zinc finger-like protein (2)	S to G	P011
2	10302	A	T	OPG021	Zinc finger-like protein (2)	K to M	P011
3	10496	C	T	OPG022	Interleukin-18-Binding protein	–	P011
4	10507	C	T	OPG022	Interleukin-18-Binding protein	V to I	P011
5	10510	T	C	OPG022	Interleukin-18-Binding protein	G to S	P011
6	36088	A	G	OPG055	Protein F11- RhoA signaling inhibitor	V to A	INDEX-AB
7	36232	A	G	OPG055	Protein F11- RhoA signaling inhibitor	–	INDEX-NL
8	36975	C	T	OPG055	Protein F11- RhoA signaling inhibitor	D to N	INDEX-AB
9	36995	T	A	OPG055	Protein F11- RhoA signaling inhibitor	K to I	INDEX-AB
10	53823	C	T	OPG071	DNA polymerase (2)	E to K	INDEX-NL; INDEX-AB; P011
11	57922	T	A	OPG077	Telomere-binding protein I1	K to M	INDEX-AB; P011
12	63599	A	C	OPG083	Viral core cysteine proteinase	F to C	P011
13	64031	G	A	OPG083	Viral core cysteine proteinase	S to F	P011

Mutation	Position	From	To	Orf	Product*	Amino acid substitutions	Samples with the mutation
14	103427	C	T	OPG123	Nucleoside triphosphatase I	–	INDEX-NL; INDEX-AB; P011
15	133828	C	T	Intergenic region	–	–	INDEX-NL
16	149818	G	A	OPG175/OPG176	Copper zinc superoxide dismutase / Bcl-2-like protein	D to N (OPG176)	INDEX-NL; INDEX-AB; P011
17	150140	C	T	OPG176	Bcl-2-like protein	S to L	INDEX-NL; INDEX-AB; P011
18	156469	G	A	Intergenic region	–	–	INDEX-NL; INDEX-AB; P011
19	164157	A	G	OPG189	Ankyrin repeat protein (25)	–	P011
20	165972	C	T	OPG190	EEV type-I membrane glycoprotein	A to V	INDEX-AB; P011
21	178303	G	A	Intergenic region	–	–	INDEX-NL
22	180308	T	C	OPG208	Serpin	L to P	INDEX-AB
23	186088	C	T	OPG210	B22R family protein - Surface glycoprotein	–	INDEX-AB; P011

*Product names based on the annotation of the reference genome NC_063383
